# Regulation of Biotransformation Systems and ABC Transporters by Benznidazole in HepG2 Cells: Involvement of Pregnane X-Receptor

**DOI:** 10.1371/journal.pntd.0001951

**Published:** 2012-12-13

**Authors:** Juan P. Rigalli, Virginia G. Perdomo, Marcelo G. Luquita, Silvina S. M. Villanueva, Agostina Arias, Dirk Theile, Johanna Weiss, Aldo D. Mottino, María L. Ruiz, Viviana A. Catania

**Affiliations:** 1 Institute of Experimental Physiology (CONICET), School of Biochemical and Pharmaceutical Sciences (UNR), Rosario, Argentina; 2 Department of Clinical Pharmacology and Pharmacoepidemiology, University of Heidelberg, Heidelberg, Germany; McGill University, Canada

## Abstract

**Background:**

Benznidazole (BZL) is the only antichagasic drug available in most endemic countries. Its effect on the expression and activity of drug-metabolizing and transporter proteins has not been studied yet.

**Methodology/Principal Findings:**

Expression and activity of P-glycoprotein (P-gp), Multidrug resistance-associated protein 2 (MRP2), Cytochrome P450 3A4 (CYP3A4), and Glutathione S-transferase (GST) were evaluated in HepG2 cells after treatment with BZL. Expression was estimated by immunoblotting and real time PCR. P-gp and MRP2 activities were estimated using model substrates rhodamine 123 and dinitrophenyl-S-glutathione (DNP-SG), respectively. CYP3A4 and GST activities were evaluated through their abilities to convert proluciferin into luciferin and 1-chloro-2,4-dinitrobenzene into DNP-SG, respectively. BZL (200 µM) increased the expression (protein and mRNA) of P-gp, MRP2, CYP3A4, and GSTπ class. A concomitant enhancement of activity was observed for all these proteins, except for CYP3A4, which exhibited a decreased activity. To elucidate if pregnane X receptor (PXR) mediates BZL response, its expression was knocked down with a specific siRNA. In this condition, the effect of BZL on P-gp, MRP2, CYP3A4, and GSTπ protein up-regulation was completely abolished. Consistent with this, BZL was able to activate PXR, as detected by reporter gene assay. Additional studies, using transporter inhibitors and P-gp-knock down cells, demonstrated that P-gp is involved in BZL extrusion. Pre-treatment of HepG2 cells with BZL increased its own efflux, as a consequence of P-gp up-regulation.

**Conclusions/Significance:**

Modifications in the activity of biotransformation and transport systems by BZL may alter the pharmacokinetics and efficiency of drugs that are substrates of these systems, including BZL itself.

## Author Summary

Chagas disease is an endemic infection caused by *Trypanosoma cruzi*. Benznidazole (BZL) is the only antichagasic drug available in most endemic countries. The liver plays a major role in disposition of endogenous and exogenous compounds and their excretion is mainly mediated by transporter proteins (such as P-gp and MRP2) that act coordinately with biotransformation enzymes (such as CYP3A4 and GST). At present there is no information on whether BZL may modulate major biotransformation systems and transporters, with potential impact on its disposition or on disposition of other therapeutic agents co-administered with BZL. BZL (200 µM) altered the expression (protein and mRNA) and activity of P-gp, MRP2, CYP3A4, and GSTπ in HepG2 cells (a cell model that retains many biochemical, morphological and functional properties of the human hepatocytes), being the nuclear receptor PXR a key mediator. Additional studies demonstrated that P-gp is involved in BZL extrusion. Alterations in the pharmacokinetics and efficiency of drugs that are substrates of these systems, including BZL itself, would be expected.

## Introduction

Chagas disease or American trypanosomiasis is an endemic infection caused by the protozoa parasite *Trypanosoma cruzi (T. cruzi)*. It is widely extended in Latin America and affects 8 million people, and at least 100 million are at risk of infection [1]. Many reports have recognized the occurrence of this zoonosis in areas where the disease is not endemic, such as the United States and Europe, mainly due to the migration of infected people [2], [3]. Currently, benznidazole (BZL, 2-(2-nitroimidazol-1-yl)-*N*-(phenylmethyl) acetamide) is the unique drug commercially available for treatment in most endemic countries. It was recently reported that in *T. cruzi* BZL is metabolized by a NADH-dependent type I nitroreductase rendering the cytotoxic and mutagenic agent glyoxal [4]. In mammalian, the nitro group is reduced to an amino group by a type II nitroreductase, with formation of free radical intermediaries and reactive oxygen species (ROS) [4]–[6]. BZL exerts its trypanocidal effect against all forms of the parasite (intra or extracellular) through these metabolites that likely bind to parasite macromolecules [7], [8].

The liver plays a major role in the elimination of endogenous and exogenous compounds. Biliary excretion of drugs is mainly mediated by members of the ATP-binding cassette (ABC) family of transporters such as P-glycoprotein (P-gp/ABCB1/MDR1), multidrug resistance-associated protein 2 (MRP2/ABCC2) and breast cancer resistance protein (BCRP/ABCG2). These transporters act coordinately with phase I and II biotransformation reactions to metabolize and excrete a wide variety of endo- and xenobiotics into bile. P-gp transports a broad diversity of lipophilic and cationic compounds including therapeutic agents and environmental pollutants [9]. MRP2 extrudes bilirubin, bile salts, carcinogens and therapeutic drugs in the form of conjugates with glutathione (GSH), glucuronic acid or sulfate [10]–[13]. BCRP transports a wide range of compounds including sulfated estrogens, anticancer drugs, antibiotics, etc [14].

The expression and activity of biotransformation systems and transporters can be altered by many factors including diet, hormones, aging, diseases, or inducing substances. Due to the co-localization and coordinated function between enzymes and transporters a simultaneous regulation of these systems has been suggested [10], [13], [15]. Regulation may occur either at the transcriptional or post-transcriptional level, resulting in changes in mRNA and protein contents, or at the level of post-translational processing [16], [17]. In general, transcriptional regulation involves ligand-activated nuclear receptors. Pregnane X-receptor (PXR, NR1I2) is a very promiscuous nuclear receptor considered the main xenosensor regulating genes involved in biotransformation and elimination of endo- and exogenous compounds. These include those of phase I enzymes (e.g. CYP3A4), phase II enzymes (e.g. glutathione S-transferase (GST)) and transporters such as P-gp and MRP2 [18], [19]. PXR functions as a defense mechanism against toxic insults, but it also constitutes the molecular basis for undesired drug-drug interactions. The drug mediated activation of PXR can accelerate its own depuration (auto-induction) or the clearance of co-administered drugs leading to reduced plasma concentrations and thus diminished efficacy of therapy. Interestingly, a study carried out in patients receiving BZL (7 mg/kg/day for 30 days, twice a day) indeed demonstrated that maximal plasma concentrations of BZL after the first dose in the morning tends to decrease with treatment time (−20% in average after 25 days of treatment) [20], suggesting the possibility of auto-induction of metabolism or absorption limiting mechanisms. At present there is no information on whether BZL truly modulates expression or activity of biotransformation systems and transporters with potential impact on its own disposition or on disposition of other therapeutic agents co-administered with BZL. In an attempt to clarify this point, in this study we explored the effect of BZL on expression and activity of the biotransformation enzymes CYP3A4 and GST classes α, μ and π, and the transporters P-gp, MRP2 and BCRP in HepG2 cells, a hepatoma cell line. The potential mediation of PXR was also evaluated.

## Materials and Methods

### Chemicals

1-chloro-2,4-dinitrobenzene (CDNB), GSH, probenecid, rhodamine 123 (Rh123), 3-(4,5-dimethylthiazol-2-yl)-2,5-diphenyltretazolium bromide (MTT), rifampicin (RIF), verapamil (VER), phenylmethylsulfonyl fluoride and leupeptin were from Sigma-Aldrich (St. Louis, MO, USA). Benznidazole was from Roche (Rio de Janeiro, Brazil). DMSO was purchased from Merck (Darmstadt, HE, Germany). All other chemicals were of analytical grade purity.

### Cell culture and treatments

The human HepG2 cell line is utilized as an approach to human hepatocytes since it is easily available and retains features that are normally lost during culturing of primary hepatocytes [21]. For example, preservation of polarity in HepG2 cells is essential for proper localization of apical membrane transporters [22]. HepG2 cells were grown in Dulbecco's modified Eagle's medium (DMEM) and Ham's F-12 medium (Invitrogen, Carlsbad, CA, USA) at a 1∶1 proportion, supplemented with 10% FBS (PAA, Pasching, Austria), 2 mM L-glutamine, a mixture of antibiotics (5 mg/ml penicillin, 5 mg/ml streptomycin and 10 mg/ml neomycin) and 0.1 mg% insulin (Invitrogen).

Cells were incubated at 37°C in a humidified atmosphere containing 5% CO_2_ as described [23]. For the treatments, unless otherwise stated, HepG2 cells were seeded in 6-well plates at a density of 5×10^5^ cells/well. BZL was dissolved in DMSO and added at different concentrations (2, 20, 200 or 1000 µM). Only DMSO was added to control cells (C). The final concentration of DMSO in the culture media was always below 0.1%. The medium was systematically renewed every 24 h.

LS180 intestinal cells were used as a model for PXR activation because these cells exhibit high PXR expression levels and are a well-known induction model [24]. Cells were grown in DMEM supplemented with 10% FBS, 2 mM L-glutamine, antibiotics (penicillin 5 mg/ml and streptomycin 5 mg/ml) and non-essential aminoacids and incubated as described for HepG2 cells.

Cell viability was assessed measuring the conversion of MTT to its formazan as described [23]. The rate of conversion in all treated groups was not statistically different from the respective control cells (data not shown).

### Western blot and real time RT-PCR studies

The effect of BZL on protein expression of biotransformation enzymes and transporters was initially assessed in cell lysates. HepG2 cells were washed twice with cold PBS and scraped with RIPA buffer (Thermo Scientific, Rockford, IL, USA) supplemented with phenylmethylsulfonyl fluoride (17 µg/ml) and leupeptin (15 µg/µl) as protease inhibitors. Lysates were passed 20 times through a 25G needle and subjected to protein concentration assay [25].

To evaluate the expression of P-gp and MRP2 at the cell surface, plasma membranes from HepG2 cells were isolated as described by Kubitz et al. [26]. Briefly, the cells were scraped in a buffer containing Tris 20 mM, sucrose 250 mM, EGTA 5 mM and MgCl_2_ 1 mM supplemented with protease inhibitors. Cell lysis was achieved through passing the cell suspension 20 times through a 25G needle, followed by protein concentration assessment [25].

Western blotting and bands quantification were performed as previously described [27]. Primary antibodies were: CYP3A4, AB1254 (Millipore, Darmstadt, HE, Germany); GSTπ, ADI-MSA-102-E and MRP2, M2III-6 (Enzo Life Sciences, Farmingdale, NY, USA); BCRP, BXP-21; glyceraldehyde-3-phosphate dehydrogenase (GAPDH, FL-335) and P-gp, H-241 (Santa Cruz Biotechnology, Santa Cruz, CA, USA); GSTYa (α class), GS-09 and GSTYb (μ class), GS23 (Oxford Biomedical Research, Rochester Hills, MI, USA) and β-actin, A-2228 (Sigma-Aldrich).

Real time RT-PCR study was performed only if alterations in protein expression were detected by western blotting. Total RNA was isolated using TRIzol reagent (Invitrogen). cDNA was synthesized from 1 µg of total RNA with the Superscript III Reverse Transcriptase (Invitrogen) using random hexamers according to manufacturer's instructions. Real time PCR reactions were carried out on a MX3000P system (Agilent Technologies, Santa Clara, CA, USA) with Platinum Taq DNA Polymerase (Invitrogen). The amount of template was quantified with SYBR Green (Invitrogen). Primers were used at a final concentration of 1 µM. Primer sequences were: MDR1(F): 5′CCAAAGACAACAGCTGAAA3′; MDR1(R): 5′TACTTGGTGGCACATAAAC3′; MRP2(F):5′AGGTTTGCCAGTTATCCGTG3′; MRP2(R): 5′AACAAAGCCAACAGTGTCCC3′; CYP3A4 (F) 5′-GTGGGGCTTTTATGATGGTCA-3′; (R) 5′-GCCTCAGATTTCTCACCAACACA-3′; GSTP1(F): 5′TATTTCCCAGTTCGAGGCCG3′; GSTP1(R): 5′TGGTACAGGGTGAGGTCTCC3′; 18S(F): 5′CGCCGCTAGAGGTGAAATTC3′; 18(R): 5′TTGGCAAATGCTTTCGCT3′. The thermocycling regime was 95°C for 2 min followed by 40 cycles of 95°C for 15 sec, 55°C for 30 sec and 72°C for 30 sec. Relative levels of MDR1, MRP2, CYP3A4 and GSTP1 mRNA normalized to 18S rRNA were calculated based on the 2^−ΔΔCt^ method [28]. Specificity of each reaction was verified with a dissociation curve between 55°C and 95°C with continuous fluorescence measure.

### Activity of transporters and biotransformation systems

The activity of P-gp in HepG2 cells was assessed measuring the intracellular content of the fluorescent compound Rh123, which inversely associates with the amount of substrate extruded [23], [29]. It is known that the probe is transported by P-gp and to some extent by BCRP [30]. To confirm P-gp participation in the efflux of Rh123, we repeated the experiments in the presence of verapamil (VER), an inhibitor of P-gp but not BCRP [31]. For this purpose, cells were cultured and treated with BZL (200 µM, 48 h) as described above. Then, treatment medium was replaced with fresh medium containing Rh123 (5 µM), with or without VER (100 µM) [32], both dissolved in DMSO. Cells were incubated for 2 h to allow the probe to enter the cells. At the end of the incubation, they were promptly washed twice with cold PBS, scraped with sucrose 0.3 M and lysed by sonication. The amount of Rh123 in the lysates was determined fluorometrically using a Multimode Detector DTX-880 (Beckman Coulter, Palo Alto, CA, USA), λexcitation = 485 nm, λemission = 535 nm.

The activity of MRP2 was determined as previously reported by Zhang et al. [33] through determination of the amount of dinitrophenyl-S-glutathione (DNP-SG) extruded by HepG2 cells into culture medium. Briefly, cells were cultured in 6-well plates and treated with BZL (200 µM, 48 h) as described above. Then, treatment medium was replaced with fresh medium containing CDNB (0.5 mM) and cells were incubated at 10°C for 30 min to allow CDNB to passively diffuse into the cytosol. In this condition, most of CDNB conversion to DNP-SG is spontaneous, i.e independent of GST activity [34]. At the end of incubation the medium was rinsed and cells were promptly washed twice with cold PBS. To evaluate the rate of DNP-SG secretion, cells were incubated with Hank's balanced salt solution at 37°C for 60 min. Samples were taken and centrifuged (3 min, 300 g, 4°C). Supernatants were treated with 10% perchloric acid and centrifuged again (2 min, 14000 g, 4°C). Remaining supernatants were used for DNP-SG detection by HPLC (Waters 600; Waters, Milford, MA, USA) as described [35]. Results are expressed as nmol of DNP-SG extruded per 10^6^ cells. To confirm MRP2 participation, probenecid (PRO, 1 mM) was added as an inhibitor [36].

To determine the effect of BZL (200 µM, 48 h) on CYP3A4 activity, cells were cultured at a density of 2×10^4^ cells/well in 96 well plates. Then, the enzyme activity was measured using the P450-Glo Luciferin-IPA CYP3A4 kit (Promega, Mannheim, BW, Germany), based on the CYP3A4 catalyzed conversion of a proluciferin substrate into a luciferin product, that can be detected in a luminometric assay using a Glomax Luminometer (Promega).

GST activity was measured according to the method of Habig et al. [37] based on the enzymatic conjugation of CDNB with GSH, thus generating DNP-SG. HepG2 cells were cultured and treated with BZL (200 µM) as already described. Then cells were harvested, lysed by sonication, and centrifuged (20 min, 10000 g, 4°C), and the supernatants were used in the assays. The reaction mixture contained PBS pH 6.50, 1 mM CDNB and 1 mM GSH. Reaction was initiated by addition of cell supernatants. Formation of DNP-SG was determined spectrophotometrically at 340 nm.

### Knock down of PXR

HepG2 cells (5×10^4^ cells/well) were seeded in 24-well plates, incubated at 37°C and subjected to transfection 24 h later. Human PXR was transiently knock down with PXR siRNA (h) (Santa Cruz Biotechnology, sc-44057) targeting the human nuclear receptor mRNA. Control siRNA-A (Santa Cruz Biotechnology, sc-37007), a non-targeting siRNA, was used as a negative control. Transfections were performed using Dharmafect4 Transfection Reagent (Dharmacon, Lafayette, CO, USA) as described [23]. Twenty four h after transfection initiation, cells were incubated with BZL at a 200 µM final concentration for 48 h. At the end of the incubation, they were rinsed, scraped and used in western blot studies as described above.

### Activity of PXR

The activation of a reporter gene under the control of a proximal sequence of *CYP3A4* gene promoter and a distal xenobiotic enhancer module (XREM), both of them containing PXR response elements is a well accepted method to quantify PXR activity [38], [39]. The plasmid pGL4.21-PXRRE-Luc was constructed as described by Gu et al. (2006) [38] with minor modifications. Human CYP3A4 proximal promoter was isolated by PCR from human genomic DNA using the primers 5′CATTGCTGGCTGAGGTGGTT3′ and 5′CATAAGCTTTGTTGCTCTTTGCTGGGCTATGTGC3′. The product was digested with BglII and HindIII and cloned into the pGL4.21 (Promega). The distal XREM was amplified with the primers 5′GGGGTACCATTCTTAGAGAGATGGTTCATTCC3′ and 5′CCGCTCGAGATCTTCGTCAACAGGTTAAAGGAG3′, digested with KpnI and BglII and cloned in the pGL4.21 plasmid already containing the proximal sequence. LS180 intestinal cells were transfected with pGL4.21-PXRRE-Luc by electroporation using the Lonza V-Kit (Lonza, Basel, Switzerland) followed by selection with Puromycin (10 µM). After successful selection, cells were seeded at a density of 30000 cells/well in 96-well plates and treated with different concentrations of BZL or RIF as a positive control for 24 h. Luminescence was measured using the Steady Glo Luciferase Assay System (Promega).

### Metabolism and transport of BZL

To estimate the amount of BZL metabolized in our experimental conditions, the amount of BZL initially added to the incubation medium was contrasted with the total amount (intracellular+extracellular) of unmodified BZL determined at the end of the incubation with 100 µM BZL for 2 h. At the end of the incubation, the medium was separated from the cells. To quantify the intracellular content of BZL, cells were lysed by sonication. Lysates were subjected to solvent extraction with acetonitrile/DMSO (1∶1) and deproteinized with 10% trichloroacetic acid. BZL was measured in supernatants by HPLC (Waters 600, Waters, Milford, MA, USA). Isocratic elution was performed with a C18 column (Luna 5 µ, Phenomenex) with a mobile phase of acetonitrile and water (2∶3, vol/vol) at a flow rate of 1.0 ml/min as described by Morilla et al. [40]. BZL was detected at 324 nm and quantified by the external standard method by the height of the peak. Culture medium was subjected to acetonitrile/DMSO extraction and deproteinization followed by assessment of BZL content, as described above.

To evaluate the possibility of BZL to be a substrate of P-gp and/or MRP2, as a first approach untreated HepG2 cells were loaded with this compound (100 µM) for 2 h, in the presence of either PRO (1 mM) or VER (100 µM). Due to its lipophilicity, it is assumed that BZL passively enters the cells. Retention of BZL into the cells after this period was inversely correlated with its extrusion. Intracellular content of BZL was determined as described above.

To further confirm the involvement of P-gp in BZL extrusion, accumulation experiments were repeated in HepG2 cells transfected with a siRNA against human P-gp (sc-29395, Santa Cruz) or with a control non-silencing siRNA (Control siRNA-A, sc-37007, Santa Cruz). Cells were seeded in 24-well plates (100000 cells/well) and 24 h later exposed to 100 nM siRNA (or control siRNA) for 48 h in the presence of Dharmafect4 (Dharmacon) as transfection reagent. Cells were further incubated for 24 h in fresh culture medium and used for BZL accumulation studies as described above.

Additionally, to assess whether BZL modulates its own excretion, cells were pre-treated with this drug (200 µM) or vehicle for 48 h and then subjected to BZL transport studies. Treated cells were washed twice with cold PBS, exposed to fresh medium containing BZL (100 µM) and VER (100 µM) or its vehicle, and further incubated for 2 h. Subsequently, BZL intracellular content was measured as described above.

### Statistical analysis

Data are presented as mean ± S.D. Statistical analysis was performed using the Student *t* test (two groups) or One-Way ANOVA followed by Newman-Keuls post hoc test (for more than two groups). Significance was set at p<0.05. Studies were performed using the GraphPad Prism 3.0 software (GraphPad Software, La Jolla, CA, USA).

In the case of PXR activation studies, PXR activation luminescence units were plotted as a function of the logarithm of agonist concentration. The curves were best adjusted to a sigmoid using the GraphPad Prism 3.0 software (GraphPad Software, La Jolla, CA, USA). The goodness of adjustments was confirmed with *R* ^2^ values, which were 0.949 and 0.954 for BZL- and RIF-treated cells, respectively.

## Results

### Effect of BZL treatment on the expression and activity of drug transporters

**Figure 1:**
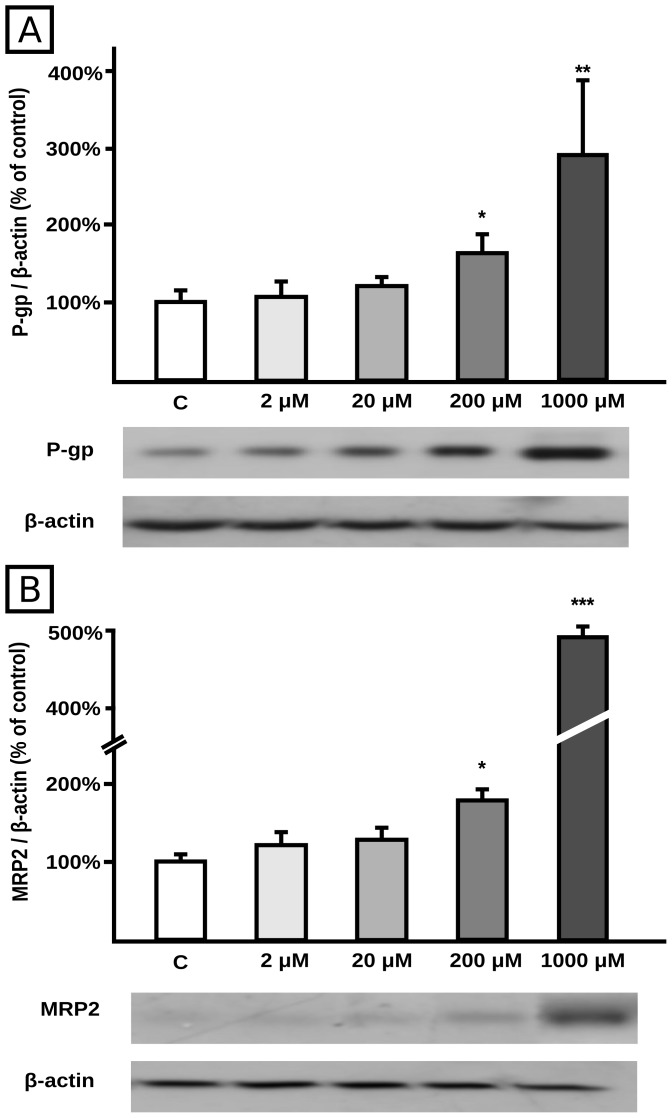
Effect of BZL on transporter expression in cellular lysates. P-gp (panel A) and MRP2 (panel B) were detected by western blotting in HepG2 total lysates after 48 h of treatment with BZL (2, 20, 200 and 1000 µM) or vehicle (C). Equal amounts of total protein (15 µg) were loaded in the gels. MRP2 and P-gp O.D. were related to β-actin O.D. Uniformity of loading and transfer from gel to PVDF membrane was also controlled with Ponceau S. The data on O.D. (% of C) are presented as mean ± S.D. (n = 3). Typical western blot detections are shown at the bottom. *Significantly different from C, p<0.05; **Significantly different from C, p<0.01; ***Significantly different from C, p<0.001.

BZL treatment for 48 h increased P-gp and MRP2 protein contents at 200 µM (+60% and +75%, respectively) and 1000 µM (+180% and +390%, respectively), with no changes at 2 or 20 µM concentrations, clearly showing a concentration-dependent effect (Fig. 1A and 1B, respectively) as detected in cell lysates. Since 1000 µM is higher than usual plasma concentrations reached during BZL treatment (30–100 µM) [41], [42], the lowest concentration (200 µM) producing a significant induction of P-gp and MRP2 was subsequently used.

**Figure 2:**
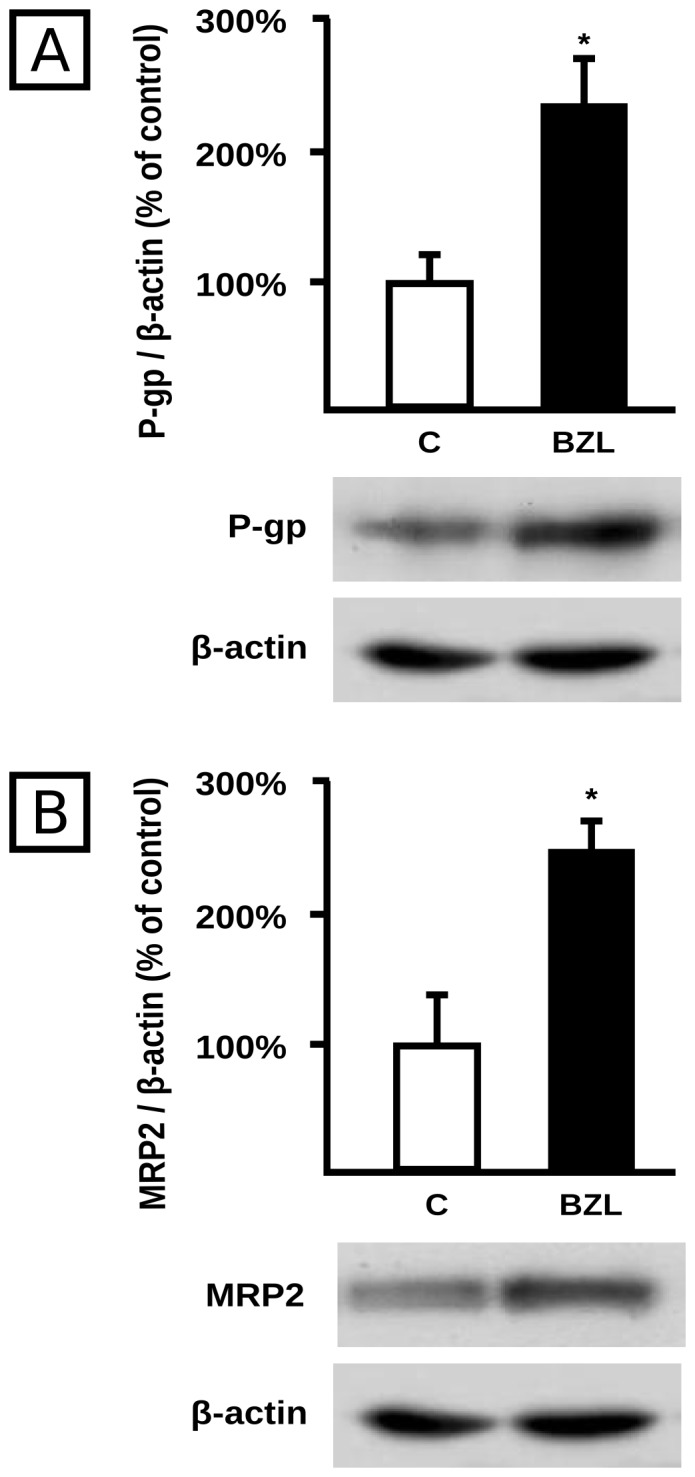
Effect of BZL on transporter expression in crude plasma membranes. P-gp (panel A) and MRP2 (panel B) were detected by western blotting in HepG2 plasma membranes after 48 h of treatment with BZL (200 µM) or vehicle (C). Equal amounts of total protein (5 µg) were loaded in the gels. MRP2 and P-gp O.D. were related to β-actin O.D. Uniformity of loading and transfer from gel to PVDF membrane was also controlled with Ponceau S. The data on O.D. (% of C) are presented as mean ± S.D. (n = 3). Typical western blot detections are shown at the bottom. *Significantly different from C, p<0.05.

P-gp and MRP2 are efflux proteins mainly localized at the plasma membrane. BZL (200 µM, 48 h) increased P-gp and MRP2 protein expression in crude plasma membranes by 138% and 150%, respectively (Fig. 2A and 2B).

To determine whether up-regulation of P-gp and MRP2 results from increased mRNA levels, we determined their expression by real time PCR. BZL treatment (200 µM, 48 h) produced a significant increase in P-gp and MRP2 mRNA levels normalized to rRNA 18S (332±151% vs 100±69% and 293±137% vs 100±57% for BZL and controls, respectively, n = 6, p<0.05), suggesting transcriptional regulation of the respective genes or stabilization of mRNA, both of which are mediated by a nuclear receptor [43], [44].

In contrast to P-gp and MRP2, BCRP protein expression was not affected by BZL treatment (200 µM, 48 h, data not shown).

**Figure 3:**
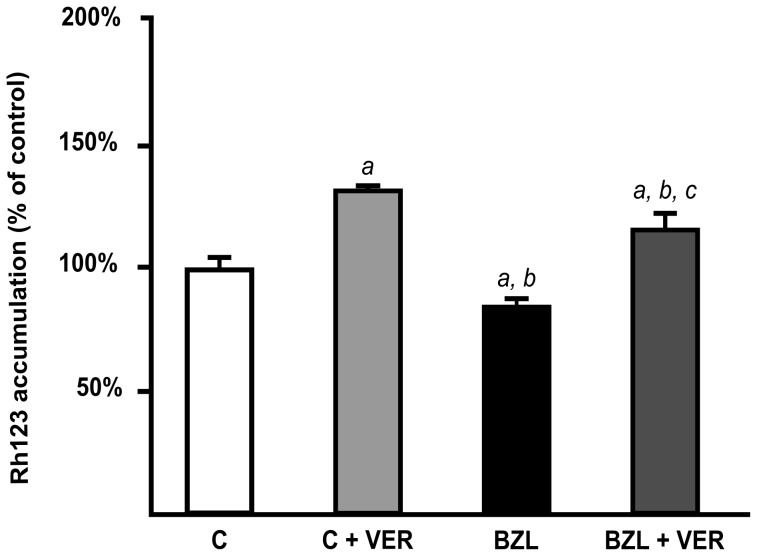
Effect of BZL on P-gp activity. Accumulation of Rh123, in the presence or absence of verapamil (VER; 100 µM), was inversely correlated with P-gp activity in cells pretreated with BZL (200 µM) or vehicle (C) for 48 h. Data are presented as percentages referred to the accumulation in C, considered as 100%, and were expressed as means ± S.D. (n = 3). *a:* significantly different from C; *b:* significantly different from C+VER; *c:* significantly different from BZL. Significance levels were set at p<0.05.

To evaluate the functional impact of P-gp and MRP2 up-regulation, we estimated their transport activities using different experimental strategies that were found optimal in each case [29], [33]. The up-regulation of P-gp by BZL indeed correlated well with a reduced intracellular content of its substrate Rh123 (−15%) when compared to control cells (Fig. 3). Intracellular level of Rh123 was increased by VER in both control and BZL-treated cells (+30% and +37%, respectively), confirming the contribution of P-gp to Rh123 efflux.

**Figure 4:**
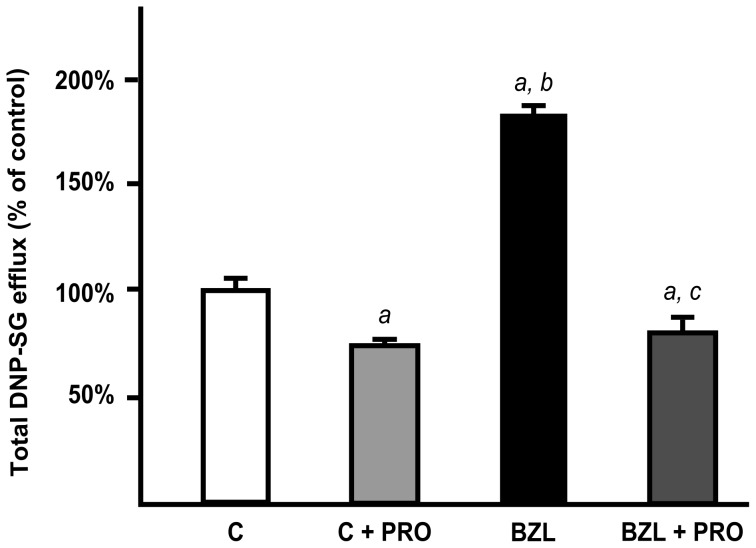
Effect of BZL on MRP2 activity. Extrusion of DNP-SG in the presence or absence of probenecid (PRO; 1 mM), was determined in supernatants of cells pretreated with BZL (200 µM, 48 h) or vehicle (C) by HPLC. Samples were taken after 60 min of incubation. Data (means ± S.D, n = 3) are presented as percentage of DNP-SG extruded in control cells. *a:* significantly different from C; *b:* significantly different from C+PRO; *c:* significantly different from BZL. Significance levels were set at p<0.05.

The excretion rate of DNP-SG in BZL-treated cells was higher (about 80%) than in control cells (Fig. 4), agreeing well with the higher content of MRP2 protein. The addition of PRO inhibited the efflux of DNP-SG both in control and BZL-treated cells (−25% and −55%, respectively), consistent with participation of a MRP transporter.

### Effect of BZL on the expression and activity of CYP3A4 and GST

**Figure 5:**
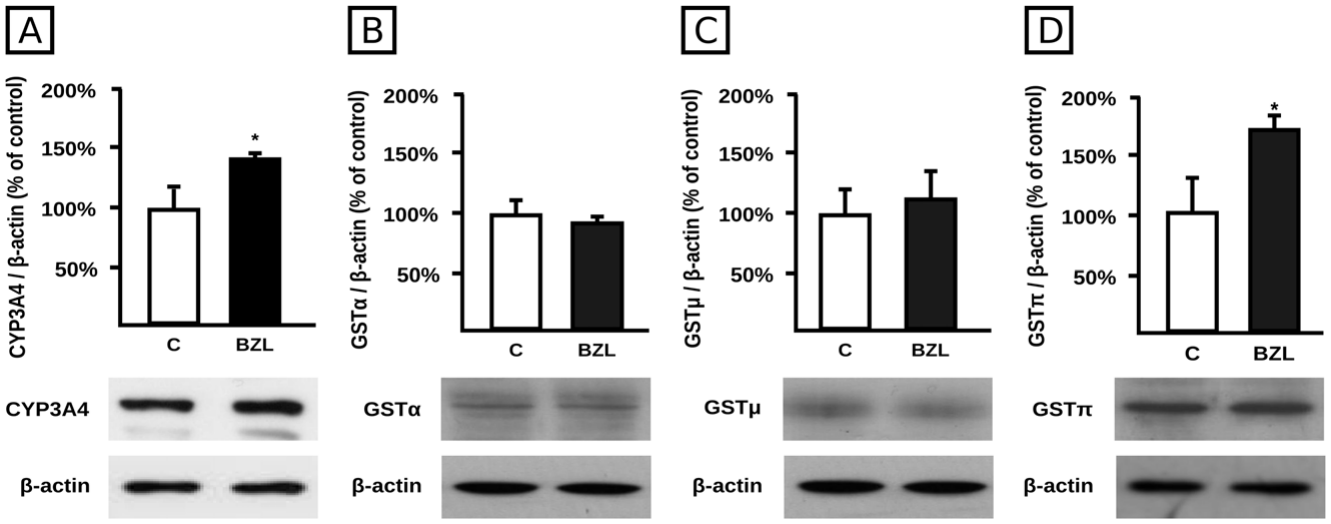
Effect of BZL on CYP3A4 and GST expression. Cells were exposed either to vehicle (C) or BZL (200 µM) for 48 h. CYP3A4 (panel A), GSTα (panel B), GSTμ (panel C), and GSTπ (panel D) levels were estimated by western blotting. Equal amounts of total protein (15 µg) were loaded in the gels. CYP3A4 or GST O.D. was related to β-actin O.D. Uniformity of loading and transfer from gel to PVDF membrane was also controlled with Ponceau S. The data on O.D. (% of C) are presented as mean ± S.D. (n = 3). Typical western blot detections are shown at the bottom. *Significantly different from C, p<0.05.

We additionally evaluated the effect of BZL treatment (200 µM, 48 h) on CYP3A4 and GST, as important biotransformation systems which usually generate substrates for P-gp and MRP2. CYP3A4 protein expression showed an induction of 43% in BZL treated cells (Fig. 5A). GSTπ class was the only GST induced by BZL (+75%, Fig. 5D). No changes were observed in expression of α or μ GSTs (Fig. 5B and 5C). A higher stability of mRNA or a transcriptional up-regulation by BZL of *CYP3A4*, and *GSTP1*, the gene that encodes for the only human isoform of GSTπ, is inferred from the Real Time PCR study, as more mRNA was detected in BZL treated cells (150±23% vs 100±21% for *CYP3A4*, n = 4, p<0.05, and 173±84% vs 100±18%, n = 7, p<0.05 for *GSTP1*).

Although an induction of CYP3A4 at protein and mRNA levels was observed, BZL decreased its activity (58±6% vs 100±4%, n = 3, p<0.05), consistent with an inhibitory action.

BZL increased GST activity towards CDNB (208±31% vs 100±8% for BZL and controls, respectively, n = 3, p<0.05), agreeing well with up-regulation of GSTπ.

### Metabolism and transport of BZL

No difference was observed between the amount of intact BZL assessed at the beginning and at the end of the incubations (data not shown), suggesting that metabolism, if any, was of minor significance in our experimental conditions (100 µM BZL, 2 h).

**Figure 6:**
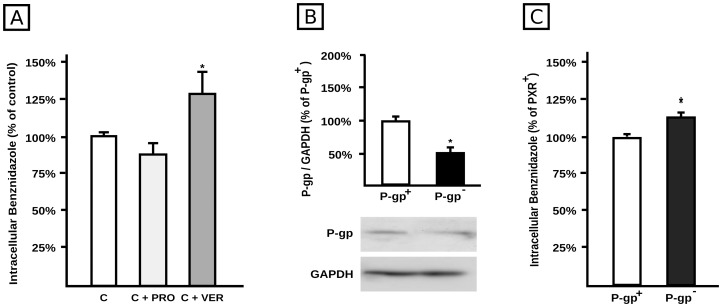
Role of P-gp in BZL transport. **A.** Confluent HepG2 cells were loaded with BZL (100 µM, 2 h) in the presence of either verapamil (VER; 100 µM) or probenecid (PRO, 1 mM). Control cells (C) were exposed to inhibitors vehicle. BZL accumulation was determined in cellular lysates by HPLC. Data (means ± S.D, n = 3) are expressed as percentage of BZL accumulated in control cells. *Significantly different from all the other groups, p<0.05. **B.** P-gp levels were estimated by western blotting in lysates from HepG2 cells transfected either with 100 nM Control siRNA-A (P-gp^+^) or with 100 nM Mdr-1 (h) si-RNA (P-gp^−^). Equal amounts of total protein (7 µg) were loaded in the gels. O.D. from P-gp was related to GAPDH O.D. Typical western blot detections from both groups are shown at the bottom. The results (% of P-gp^+^ cells) are expressed as mean ± S.D. (n = 3). *Significantly different from P-gp^+^, p<0.05. **C.** P-gp^+^ and P-gp^−^ cells were loaded with BZL (100 µM, 2 h). BZL accumulation was determined in cellular lysates by HPLC. Data (means ± S.D., n = 4) are expressed as percentage of BZL accumulated in P-gp^+^ cells. *Significantly different from P-gp^+^.

To elucidate if P-gp and/or MRP2 are involved in BZL efflux, HepG2 cells were incubated with BZL (100 µM) in the presence or absence of VER (100 µM) or PRO (1 mM), for 2 h. At the end of incubation, intracellular concentration of BZL was measured by HPLC. As shown in Fig. 6A the intracellular accumulation of BZL was not modified by PRO, excluding participation of MRP2 or other MRPs as potential BZL transporters. In contrast, intracellular accumulation was higher in cells exposed to VER (+28%), suggesting that P-gp was at least partially involved in the efflux of the drug. To further confirm this assumption, BZL intracellular accumulation was measured in P-gp knock down cells (P-gp^−^) or in cells transfected with a non-silencing RNA (P-gp^+^). P-gp^−^ cells exhibited a diminished P-gp expression (−50%) (Fig. 6B), and increased BZL accumulation (+15%, Fig. 6C), strongly suggesting participation of P-gp in BZL transport. As a positive control, accumulation of Rh123 was increased by 70% in P-gp^−^ cells (n = 4, p<0.05).

**Figure 7:**
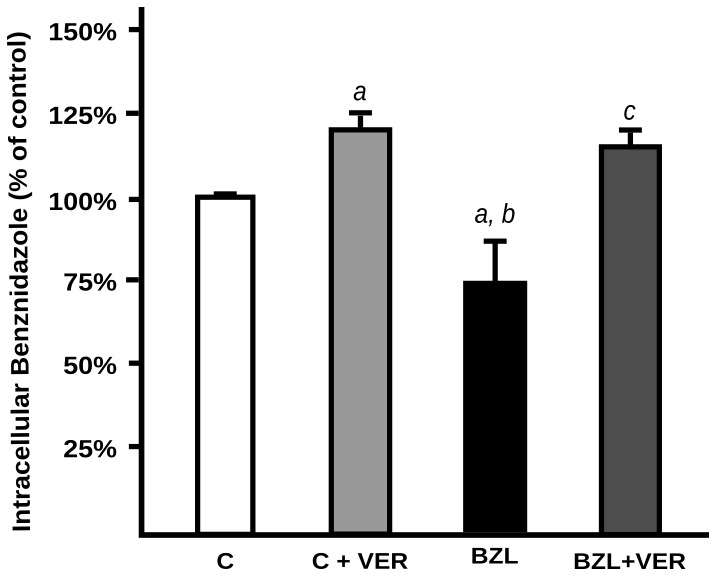
Effect of BZL on its own transport. HepG2 cells were pretreated with BZL in conditions shown to induce P-gp expression (200 µM, 48 h) or (C) vehicle. Then they were loaded with BZL (100 µM, 2 h) with or without verapamil (VER; 100 µM). BZL accumulation was determined in cellular lysates by HPLC. Data (means ± S.D, n = 3) are expressed as percentage of BZL accumulated in control (C) cells. *a:* significantly different from C; *b:* significantly different from C+VER; *c:* significantly different from BZL, p<0,05.

When HepG2 cells were pretreated with BZL (200 µM, 48 h) or vehicle, and further incubated with BZL (100 µM, 2h) for BZL transport studies, its intracellular accumulation was significantly lower in pretreated cells (−27%), indicating increased efficiency of drug extrusion. The addition of VER (100 µM) abolished this difference (Fig. 7) suggesting that P-gp induction was responsible for the increased excretion of BZL in cells pretreated with this same drug.

### Mediation of BZL effects by nuclear receptors

**Figure 8:**
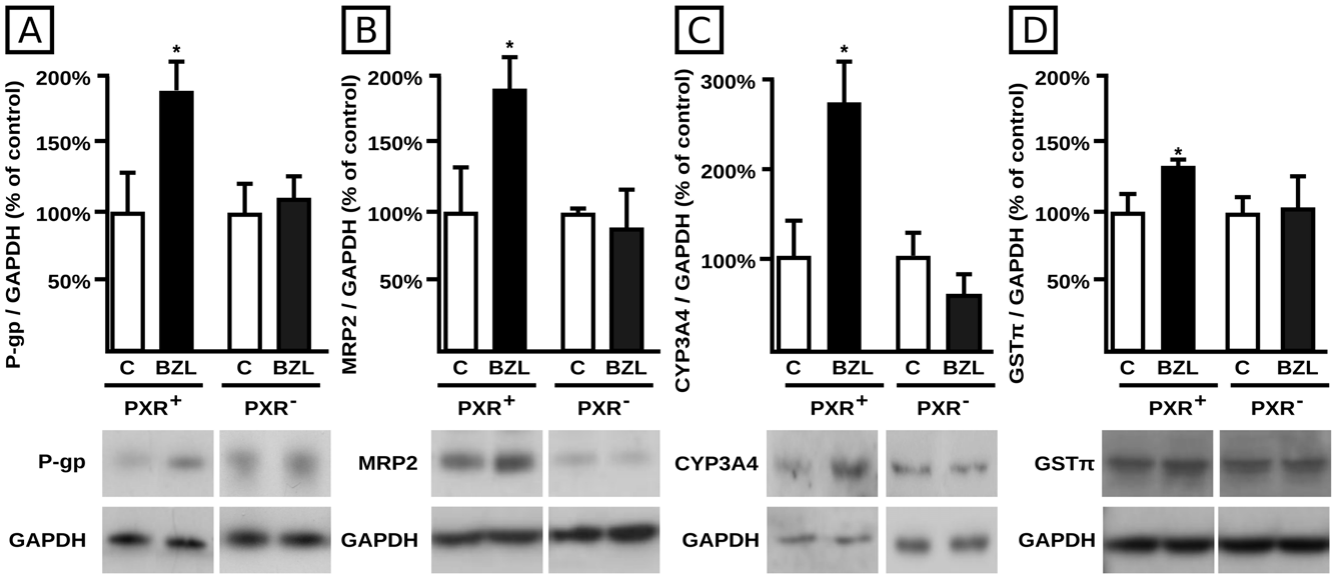
Effect of PXR knock down on BZL mediated P-gp, MRP2, CYP3A4 and GSTπ induction. P-gp (panel A), MRP2 (panel B), CYP3A4 (panel C) and GSTπ (panel D) levels were estimated by western blotting in lysates from HepG2 cells transfected either with 100 nM Control siRNA-A (PXR^+^) or 100 nM PXR siRNA (h) (PXR^−^) and exposed to BZL (200 µM, 48 h) or vehicle (C). Equal amounts of total protein (7 µg) were loaded in the gels. O.D. from each protein was related to GAPDH O.D. Uniformity of loading and transfer from gel to PVDF membrane was also controlled with Ponceau S. Typical western blot detections from each group are shown at the bottom of bar graphics. The results (% of each control) are expressed as mean ± S.D. (n = 3). *Significantly different from C, p<0.05.

Given that PXR is a key mediator in the co-regulation of drug metabolism and transport by xenobiotics, it was of interest to evaluate if the effects of BZL on P-gp, MRP2, CYP3A4 and GST were associated with the activation of PXR. We used a siRNA-driven mechanism to knock down its expression. Using this same strategy we previously observed a significant decrease in PXR expression (−75%) in PXR^−^ cells when compared to cells exposed to a non-targeting siRNA (PXR^+^) [23]. P-gp, MRP2, CYP3A4 and GST expression in control cells was set at 100%. PXR^+^ cells exhibited induction of P-gp (Fig. 8A), MRP2 (Fig. 8B), CYP3A4 (Fig. 8C) and GSTπ (Fig. 8D) by BZL (+86%, +88%, +178%, and +31%, respectively) similar to that previously observed in wild-type cells. The siRNA-mediated PXR knock down completely abolished BZL-mediated induction for these proteins, strongly suggesting that this nuclear receptor is indeed implicated in the regulation of human *MDR1*, *MRP2*, *CYP3A4* and *GSTP1* genes by this drug (Fig. 8A, 8B, 8C and 8D, respectively).

**Figure 9:**
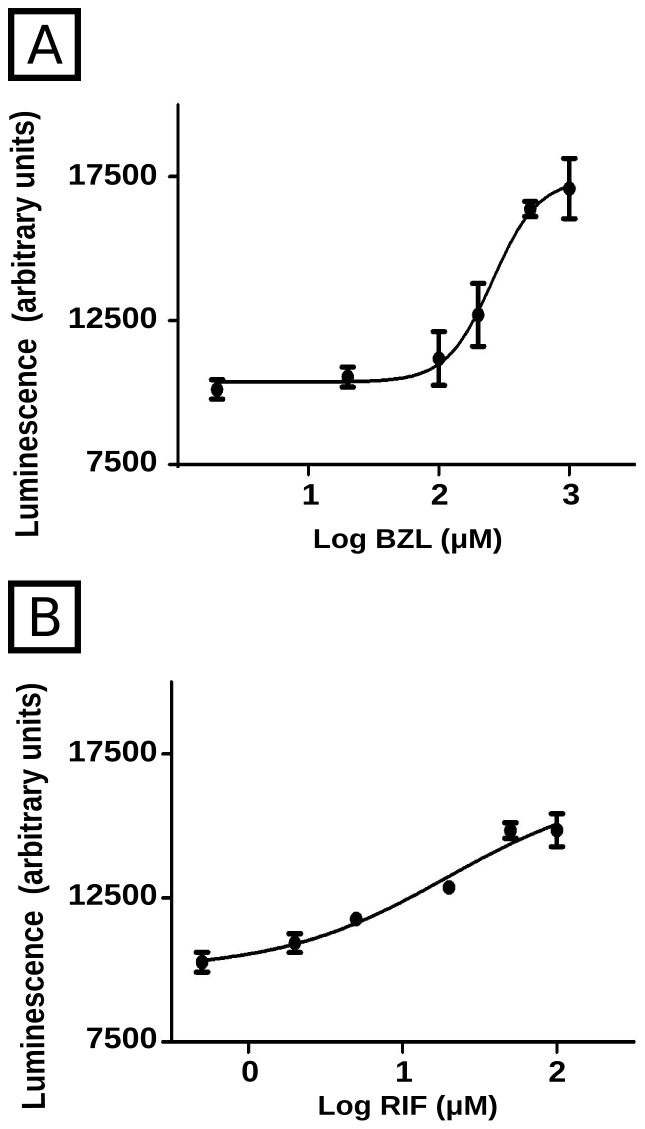
BZL-mediated activation of PXR. PXR activation was measured through the activation of the firefly luciferase gene under control of two PXR responsive elements after treatment with different concentrations of BZL (panel A) or RIF as positive control (panel B).

PXR activation by BZL was measured using a reporter system, in which RIF was used as a positive control. Results show that BZL was indeed able to activate PXR, being the calculated EC_50_ 259±38 µM (Fig. 9).

## Discussion

The interplay between biotransformation systems and drug transporters and its significance in drug disposition is well recognized [45]. Using the HepG2 cell model, we evaluated the effect of the antichagasic drug BZL on expression and activity of CYP3A4 and GST biotransformation systems and MRP2 and P-gp transporters, as representatives of major drug eliminating systems.

Data on CYP3A4 shows an increase in its protein and mRNA expression after treatment with BZL, with no concomitant increase in its enzyme activity. In contrast, BZL even significantly decreased CYP3A4 activity. Masana et al. [46] reported that acute administration of BZL to rats (30 mg/kg, i.p.) prolongs pentobarbital induced sleeping time. This effect was attributed to the inhibition of the hepatic microsomal biotransformation systems aminopyrine and ethylmorphine N-demethylases (phase I reactions), consequence of their covalent interactions with BZL electrophilic metabolites (non-competitive inhibition). It is possible that a similar phenomenon occurred in our model as a consequence of post-translational regulation of CYP3A4. This isoform is the most abundant CYP450 enzyme expressed in human liver, metabolizing over 50% of therapeutic drugs [47]. Whether administration of BZL to chagasic patients results in suppression of hepatic CYP3A4 activity is not known. If so, a significant influence on the disposition of a wide range of co-administered drugs can be expected.

Regarding Phase II systems, GSTs catalyze the conjugation of GSH to electrophilic substrates, which are often products of oxidative phase I metabolism. Here we report up-regulation of GST activity towards CDNB by BZL, agreeing well with up-regulation of GSTπ protein and GSTP1 mRNA. Hepatic GSTπ is predominantly expressed in ductular epithelial cells under physiological conditions, and is hardly expressed in parenchymal cells [48]. GSTs, particularly GSTP1, conjugate and protect against the cytotoxic effects of endogenous and exogenous electrophilic agents. In *T. cruzi* BZL is metabolized by a NADH-dependent type I nitroreductase rendering the cytotoxic and mutagenic agent glyoxal, and this is tentatively linked to its antiparasitic action, whereas in mammals, the nitro group is reduced to an amino group by a type II nitroreductase, with formation of free radical intermediaries [4]. Augmented hepatic GST activity by BZL pre-treatment could additionally contribute to neutralize electrophilic derivatives from BZL itself, thus protecting the liver cells from potential deleterious effects.

More efficient detoxification of endo- and xenobiotics is also associated with higher levels of efflux transporter proteins such as P-gp and/or MRPs. As a most significant finding, we observed a concentration-dependent effect of BZL on the expression of both P-gp and MRP2 potentially leading to changes of pharmacokinetics of co-administered drugs. The auto-inducer effect of BZL could additionally modify its own disposition as suggested by the experiments of BZL transport in BZL pre-treated cells. These cells showed a decreased intracellular accumulation of BZL, partially reversed by VER, a P-gp inhibitor. The contribution of P-gp to BZL transport was further confirmed in P-gp knock down cells. However, accumulation of a model P-gp substrate was more affected than BZL in P-gp^−^ cells. In consequence, it is likely that additional transporters are also involved in BZL efflux. MRP2 induction by BZL could lead to alterations in the disposition of co-administered drugs, substrates of this transporter.

Whether the current findings on induction of biotransformation and transport systems also occur in patients receiving BZL is not known. The doses used for the treatment of Chagas disease (5–10 mg/kg body weight, administered for 30–60 days) lead to plasma concentrations varying from 30 to 100 µM [41], [42], which are slightly below the lowest concentration used in this study showing inductive properties, i.e. 200 µM. In unpublished experiments, we observed that exposure of HepG2 cells to 100 µM BZL induced CYP3A4, GSTπ, P-gp and MRP2 as detected by western blotting. As the treatment period of chagasic patients is between 30 to 60 days (or even up to five months in case of disease reactivation), an effect of BZL in humans cannot be ruled out since our experimental approach only covered a few days. In addition, plasma levels of BZL higher than the concentrations currently used could be reached in chagasic patients under pre-operative procedures for cardiac transplantation, since doses of BZL 4- to 5-fold higher than regular ones are used [49].

P-gp is significantly expressed at the apical membrane of enterocytes limiting the absorption of respective substrates [9]. BZL is orally administered and in consequence, intestinal induction of P-gp expression or activity would affect its absorption. Additional experiments in Caco-2 cells, a cell line used as a model for intestinal human epithelium, showed that BZL (200 µM, 48 h) increased protein expression of P-gp, CYP3A4, GSTπ, and MRP2 to a similar extent as found in HepG2 cells (unpublished results). Interestingly, Raaflaub reported that the maximal plasma concentrations in patients receiving BZL for 30 days (7 mg/kg/day) tend to decrease with the course of treatment [20]. Taken together the data from the current study suggest the possibility of a progressive decrease in BZL absorption and/or increase in BZL metabolism/elimination after its therapeutic administration. Unfortunately, we found no studies in the literature evidencing this possibility, or a link with decreased therapeutic efficacy. Experiments in animals, evaluating the effect of BZL on hepatic vs intestinal systems after in vivo administration, could represent an approach to overcome these questions.

Results demonstrating that the knock down of PXR prevents the induction of P-gp, MRP2, CYP3A4 and GSTπ in HepG2 cells, indicate that this nuclear receptor is causally involved as a mediator. In addition, using a reporter gene assay we demonstrated PXR activation by BZL (Fig. 9). This is the first study reporting PXR activation by BZL and mediation of BZL effects. We found that BZL does not modulate BCRP protein expression, which is consistent with preferential regulation of BCRP by other factors, rather than by PXR [14]. Ketoconazole, a recognized PXR antagonist and CYP3A4 inhibitor, has demonstrated *in vitro* activity against *T. cruzi* [7], [50]. A higher cure rate was observed when infected mice were treated with a combination of BZL and ketoconazole in comparison with those treated with ketoconazole or BZL alone [51]. A synergistic effect was proposed as an explanation. It is also possible that ketoconazole increases exposure to BZL by inhibiting CYP3A4, PXR, and P-gp [52]. This evidence would imply changes in BZL pharmacokinetics and consequently could lead to a higher time of drug-parasite contact, thus improving efficacy of treatment.

Because PXR knock down procedure totally prevented BZL mediated induction of biotransformation and transporter systems (Fig. 8), these results unambiguously demonstrate PXR's high relevance. However, influence of nuclear receptors other than PXR cannot be entirely ruled out. This might especially come to the fore in different experimental conditions, e.g. involving different treatment protocols, cell models, etc. Moreover, in conditions of altered cellular redox status, ROS modulate these same systems via the nuclear factor erythroid 2-related factor 2 [53]. BZL is in turn known to stimulate ROS formation in a dose-dependent manner [6].

Other antiparasitic drugs have shown to modulate biotransformation and transporter genes with important impact on drug disposition. On this regard, Bapiro et al. [54] demonstrated that quinine and albendazole induced CYP1A1 and CYP1A2 in HepG2 cells at concentrations equivalent to those achieved in therapeutic protocols alerting about the risk of combining quinine or albendazole with other drugs that are metabolized by these systems. During antimalarial treatment with artemisinin, disease reactivation during monotherapy was associated with decreased artemisinin plasma levels [55]. The authors postulate that artemisinin induces its own elimination probably by increasing first pass metabolism. More recently, Burk et al. [56] demonstrated that LS174T cells and primary human hepatocytes treated with artemisinin showed specific selective induction of CYP2B6, CYP2C19, CYP3A4 and MDR1 mRNA expression mediated by activation of PXR and constitutive androstane receptor. Antiparasitic drugs can also modulate biotransformation enzymes or ABC transporters in parasites, thus leading to increased resistance to treatment. On this regard, Portal et al. [57] and Murta et al. [58] reported on development of BZL resistance in *T. cruzi* as a consequence of changes in parasitic cytochrome P450 enzyme or P-gp activities.

In conclusion, our data demonstrate the simultaneous induction of P-gp, MRP2, CYP3A4 and GSTπ expression by BZL mediated through increased activity of PXR. These findings suggest a potential impact of BZL administration on the pharmacokinetics of BZL itself (auto-induction) and of compounds that are eliminated by these biotransformation and excretion systems.
